# Elevated proportion of TLR2- and TLR4-expressing Th17-like cells and activated memory B cells was associated with clinical activity of cerebral cavernous malformations

**DOI:** 10.1186/s12974-022-02385-2

**Published:** 2022-02-02

**Authors:** Camilla Castro, Hugo A. A. Oyamada, Marcos Octávio S. D. Cafasso, Lana M. Lopes, Clarice Monteiro, Priscila M. Sacramento, Soniza Vieira Alves-Leon, Gustavo da Fontoura Galvão, Joana Hygino, Jorge Paes Barreto Marcondes de Souza, Cleonice A. M. Bento

**Affiliations:** 1 Department of Microbiology and Parasitology, Federal University of the State of Rio de Janeiro, Frei Caneca 94, Rio de Janeiro, RJ 20261-040 Brazil; 2 Post-Graduate Program in Microbiology, University of the State of Rio de Janeiro, Rio de Janeiro, Brazil; 3 Post-Graduate Program in Neurology, University of the State of Rio de Janeiro, Rio de Janeiro, Brazil; 4 Translational Neuroscience Laboratory (LabNet), University of the State of Rio de Janeiro, Rio de Janeiro, Brazil; 5 Service of Neurosurgery, University Hospital of the Federal University of Rio de Janeiro, Rio de Janeiro, RJ Brazil; 6 Post-Graduate Program of Surgical Sciences, Federal University of Rio de Janeiro, Rio de Janeiro, Brazil

**Keywords:** Cerebral cavernous malformations, T cells, Th17 cells, TLR, Cytokines, B cells, LPS

## Abstract

**Background:**

Recent evidences have suggested the involvement of toll-like receptor (TLR)-4 in the pathogenesis of cerebral cavernous malformations (CCM). Elevated frequency of TLR^+^T-cells has been associated with neurological inflammatory disorders. As T-cells and B-cells are found in CCM lesions, the objective of the present study was to evaluate the cytokine profile of T-cells expressing TLR2 and TLR4, as well as B-cell subsets, in asymptomatic (CCM_Asympt_) and symptomatic (CCM_Sympt_) patients.

**Methods:**

For our study, the cytokine profile from TLR2^+^ and TLR4^+^ T-cell and B-cell subsets in CCM_Asympt_ and CCM_Sympt_ patients was investigated using flow cytometry and ELISA. T-cells were stimulated in vitro with anti-CD3/anti-CD28 beads or TLR2 (Pam3C) and TLR4 (LPS) ligands.

**Results:**

CCM_Symptc_ patients presented a higher frequency of TLR4^+^(CD4^+^ and CD8^+^) T-cells and greater density of TLR4 expression on these cells. With regard to the cytokine profile, the percentage of TLR2^+^ and TLR4^+^ Th17 cells was higher in CCM_Sympt_ patients. In addition, an elevated proportion of TLR4^+ ^Tc-1 cells, as well as Tc-17 and Th17.1 cells expressing TLR2 and TLR4, was observed in the symptomatic patients. By contrast, the percentage of TLR4^+^ IL-10^+^CD4^+^ T cells was higher in the CCM_Asympt_ group. Both Pam3C and LPS were more able to elevate the frequency of IL-6^+^CD4^+^T cells and Th17.1 cells in CCM_Sympt_ cell cultures. Furthermore, in comparison with asymptomatic patients, purified T-cells from the CCM_Sympt_ group released higher levels of Th17-related cytokines in response to Pam3C and, mainly, LPS, as well as after activation via TCR/CD28. Concerning the B-cell subsets, a higher frequency of memory and memory activated B-cells was observed in CCM_Sympt_ patients.

**Conclusions:**

Our findings reveal an increase in circulating Th17/Tc-17 cell subsets expressing functional TLR2 and, mainly, TLR4 molecules, associated with an increase in memory B-cell subsets in CCM patients with clinical activity of the disease.

**Supplementary Information:**

The online version contains supplementary material available at 10.1186/s12974-022-02385-2.

## Introduction

Cerebral cavernous malformations (CCM), or cavernous angiomas, are vascular lesions consisting mainly of clusters of grossly dilated brain capillaries presenting altered permeability due to the loss of tight junctions and muscular and elastic tissues, which predisposes patients to increased risk of hemorrhagic stroke, epilepsy and other neurological disorders [1]. CCMs are among the most common vascular malformations of the Central Nervous System (CNS) with an estimated prevalence ranging from 0.4 to 0.9%, mostly detected in adolescents and young adults [2, 3]. Although the majority of CCM cases are sporadic, approximately 20% are inherited as an autosomal dominant trait and, different from sporadic cases, the inherited disease manifests with multiple lesions [1, 4]. In both sporadic and familial cases, CCM results from a mutation at one of three CCM gene loci, CCM1/Krit1, CCM2/MGC4607, and CCM3/PDCD10. These genes encode components of a heterotrimeric intracellular adaptor protein complex, named CCM complex [2–5], that stabilizes the junctions of endothelial cells by inhibiting overactivation of the MEKK3–KLF2/4 signaling pathway [6, 7]. Despite the knowledge of CCM complex involvement, the severity and natural course of the disease are highly variable, even in patients carrying the same genetic mutations [1–3], suggesting the existence of others genetic/epigenetic factors and/or environmental disease modifiers, some of which involving inflammatory mediators, that affect disease outcomes, probably by activating the MEKK–KLF2/4 pathway [8, 9].

The risk of symptomatic CCM has been associated with elevated plasma levels of the pro-inflammatory cytokines interleukin (IL)-1β, IL-2, interferon (IFN)-γ, tumor necrosis factor (TNF)-α and lower concentrations of the anti-inflammatory cytokine IL-10 [10, 11]. In addition, gene polymorphisms expected to increase the expression of membrane CD14 (mCD14) and toll-like receptor (TLR)-4 have been correlated with more severe CCM disease [10, 11]. The mCD14/TLR4 complex forms a pattern recognition receptor classically expressed on monocytes, which is responsible for the recognition of lipopolysaccharides (LPS), a pathogen-associated molecular pattern (PAMP) from Gram-negative bacteria (GNB). Upon LPS engagement, the CD14/TLR4, in association with myeloid differentiation primary response protein 88 (MyD88), activates nuclear factor (NF)-κB, promoting the production of pro-inflammatory cytokines [12, 13]. Interestingly, in experimental model of the disease, LPS, by activating TLR4–MEKK3–KLF2/4 signaling in endothelial cells, accelerated CCM formation in the brain of those animals [14]. Although this study have demonstrated that LPS-induced brain CCM lesions did not depend on were a phenomenon independent of local immune cells, macrophages, B cells and (CD4^+^ and CD8^+^) T cells have been observed in perivascular regions of CCM lesions in patients [14]. Moreover, immunocomplexes consisting of IgG and terminal products of complement activation were also detected in CCM lesions [6]. Interestingly, analysis of the complementarity-determining regions 3 (CDR3) sequence of the variable domains of these IgG revealed a local Ig production containing somatic hypermutation into CDR3 regions [6], a genetic event classically dependent on CD4^+^ T cell help [7, 15].

According to the pattern of secreted cytokines, human effector CD4^+^ T cells are divided into three major subsets, Th1 (IFN-γ^+^), Th2 (IL-4^+^) and Th17 (IL-17^+^). Th1 cells not only favor macrophage activation by releasing IFN-γ, but also assist CD8^+^ T lymphocytes differentiation into cytotoxic cells able to kill target cells through the release of perforin and granzymes [16, 17]. Concerning the cytokine pattern, activated CD8^+^ T cells can be named Tc-1 (IFN-γ^+^) [18] or Tc-17 (IL-17^+^) [19]. The signature cytokines produced by Th2 (IL-4) and Th17 (IL-17) cells are implicated in inflammatory processes involving eosinophil and neutrophil activation, respectively [17, 20, 21]. With regard to adaptive humoral immune response, the production of IgG, IgA and IgE neutralizing antibodies classes by activated B cells, and the generation of memory B cells, are dependent on follicular helper T cells (T_FH_), a CD4^+^ T cell phenotype identified by the high expression of surface CXCR5 and production of the signature cytokine IL-21 [7, 15]. Although these lymphocytes have been implicated in protection against different pathogens, some effector T cell subsets, mainly those expressing TLR2 and TLR4, are involved in the severity of neurological inflammatory disorders, such as neuromyelitis optic spectrum disorders (NMOSD) [22, 23] and the multiple sclerosis (MS) [24, 25], both autoimmune demyelinating diseases of the CNS. Despite high T cell numbers in CCM lesions, no study has yet been designed to analyze the cytokine profile of these lymphocytes, particularly among T cell subsets expressing TLRs. It is possible that PAMPs may adversely impact the outcome of CCM by favoring the activation of pathogenic TLR^+^ T cells. Therefore, in the present study, we aimed to evaluate the frequency and cytokine profile of circulating CD4^+^ and CD8^+^ T cell subsets expressing TLR2 and TLR4 in asymptomatic and symptomatic CCM patients. Also, we analyzed B cell subsets.

## Methodology

### Patients

This transversal study included 37 CCM patients, 14 asymptomatic [CCM_Asympt_, mean age ± SD, 45 ± 13.1 years (7 familial/multifocal, 7 sporadic/solitary)] and 23 symptomatic [CCM_Sympt_, mean age ± SD, 40 ± 13.6 years (3 familial/multifocal, 17 sporadic/solitary)] from the Federal University of Rio de Janeiro and Brazil Cavernoma Alliance. The majority of CCM patients were women (n = 25/37; 10 asymptomatic and 15 symptomatic). Developmental venous anomaly (DVA) was identified in 03 asymptomatic and 09 symptomatic patients. Among symptomatic patients, the clinical manifestations were lesional hemorrhages (87%, n = 20) and seizure crisis (13%, n = 03). We excluded patients who had undergone partial or complete CCM lesion resection or any prior brain irradiation. Except for two patients with epilepsy, who were using Oxcarbazepine (Trileptal) to control their seizure crisis, all other CCM patients recruited were not taking any medication. As the control group (Ctrl), 20 healthy individuals with normal brain Magnetic resonance imaging (MRI) images (10 women and 10 men) (mean age ± SD, 40.1 ± 11.7 years) were also recruited from Federal University of the State of Rio de Janeiro. Subjects with a history of autoimmune disease, neoplasms, smoking, or treatment with psychotropic drugs were excluded, as well as pregnant women. The Ethics Committee for Research on Human Subjects at the Federal University of the State of Rio de Janeiro (UNIRIO, CAAE 69409617.9.0000.5258) approved the study and blood was collected only after written informed consent was obtained from each individual.

### Flow cytometry

Mouse anti-human monoclonal antibodies (mAbs) directed against CD3-PerCpCy5.5 (OKT3 clone), CD4-SB600 (SK-3 clone), CD8-FITC (3B5 clone), CD14-APC (61D3 clone), CD16-PE (CB16 clone), CD62L-FITC (Dreg-56 clone), CD45-PE (2D1 clone), CD45RO-PE-Cy7 (UCHL1 clone), TLR2-PE (TL2.1 clone), TLR4-PE (HTA125 clone), CD19-APC (HIB19 clone), CD27-FITC (CLB-27/1 clone), CD38-PE (HIT2 clone), IL-6-PE-Cy7 (MQ2-13A5 clone), IL-10-eFluor450 (JESE-9D7 clone), IL-17-Alexa Fluor 647 (N49-653 clone) and IFN-γ-Alexa Fluor780 (4S.B3 clone), and all isotype control antibodies were purchased from eBioscience (Thermo Fischer scientific, USA). To identify different cytokine-producing T cells subsets [Th1 (CD3^+^/CD4^+^ INF-γ^+^), Tc-1 (CD3^+^/CD8^+^ INF-γ^+^), Th17 (CD3^+^/CD4^+^ IL-17^+^), Th17.1 (CD3^+^/CD4^+^ INF-γ^+^IL-17^+^), Tc-17 (CD3^+^/CD8^+^ IL-17^+^) and Treg-like cells (CD3^+^CD4^+^IL-10^+^], capable of expressing TLR2 or TLR4, as well as B cells (CD19, CD27, CD38 and IL-10), whole peripheral blood (1 mL) of CCM patients was cultured in a 24-well flat microtiter plate in the absence of stimulus or in the presence of TLR2 ligand [synthetic triacylated lipopeptide (Pam3Csk4—1 μg/ml)] (InvivoGen, San Diego, CA, USA) or TLR4 agonist [LPS (100 ng/ml) from *Escherichia coli*] (Sigma-Aldrich, St. Louis, MO, USA)]. The TLR concentrations were established in studies conducted by Voo et al. [26]. The cell cultures were kept for 24 h at 37 °C in a humidified 5% CO2 incubator. For cytokine measurement optimization, brefeldin A (10 μg/mL; Sigma-Aldrich) was added 4 h before cell staining with mAbs. Briefly, whole blood cells were incubated with various combinations of mAbs for surface markers (CD3, CD45R0, CD14, CD16, CD4, CD8, TLR2, TLR4, CXCR5, CD19, CD27 and CD38) for 30 min at room temperature in the dark, according to manufacturer's instructions. The cells were washed with PBS + 2% FBS, and then, the whole blood cells were lysed with Fix/Lyse solution (BD Pharmigen, San Diego, USA) for 10 min at room temperature followed by cell permeabilization, which was performed by incubating cells in Cytofix/Cytoperm solution (BD PharMingen, San Diego,) at 4 °C for 20 min. After washing, the mAbs for intracellular cytokine staining (IL-6, IL-10, IL-17, IFN-γ and IL-21) were added in different combinations and incubated for 30 min at 4 °C. To prevent non-specific staining of cells expressing FcRs, FcR blocking reagent was used (Miltenyi Biotec, cat. 130.059.901). The cells were acquired on Attune NxT flow cytometers (Thermo Fisher Corporation) and analyzed using FlowJo software. Isotype control antibodies and single-stained samples were used to periodically check the settings and gates on the flow cytometer. After the acquisition of 100,000–300,000 events, lymphocytes were gated based on forward and side scatter properties after the exclusion of dead cells, using propidium iodide, and doublets.

### T cell cultures, cell proliferation and cytokine production

Peripheral blood mononuclear cells (PBMC) from healthy subjects and CCM patients were separated by a Ficoll–Paque gradient, and then submitted to negative selection using magnetic columns to obtain T cells according to manufacturer’s instructions (EasySep ™, cat. 17951, StemCell Technology ™, Vancouver, Canada). Briefly, 50 µL of a cocktail containing monoclonal antibodies directed against B cells, monocytes, dendritic cells and NK cells were added to a 15 mL tube containing approximately 5 × 10^7^ PBMC/mL. After 5 min of incubation, 40 µL/mL of magnetic beads were added to the cell suspension. After rapidly mixing, the cell suspension was incubated at room temperature for 5 min. Subsequently, 4 mL of HBSS were added to the cell suspension and, the tube was placed on the magnet for 5 min. Finally, the supernatants were recovered. The purity of the CD3^+^ cells was > 98%, as measured by flow cytometry (data not shown). Enriched T cell cultures were then maintained with RPMI-1640 medium supplemented with 2 μM of L-glutamine (GIBCO, Carlsbad, CA, USA), 10% fetal calf serum, 20 U/mL of penicillin, 20 μg/mL of streptomycin and 20 mM of HEPES buffer. In some cultures, Pam3Csk4 (Pam3C, 1 μg/mL) or LPS (100 ng/mL) was added. As a positive control, some T cell cultures were stimulated with anti-CD3/anti-CD28 beads (10 μl/mL). All cell cultures were kept for 48 h at 37 °C and 5% CO_2_ before evaluation of T cell proliferation and cytokine release. This culture duration was established by our group from time-course T cell proliferation analyzes in response to LPS and Pam3C (data not shown).

The T cell proliferation was measured by [^3^H] thymidine incorporation added to cultures at 4 μCi/well 8 h prior to the conclusion of the 2-day incubation period. The cells were harvested in glass fibre 8 filters in an automatic cell harvester (Beckman Coulter L56500) and radioactive incorporation was measured using a liquid scintillation counter. The results were shown as stimulation indexes (SI), that is, the mean count per minute (cpm) of stimulated T cells was divided by the mean cpm of unstimulated T cells from the same patient group. An SI > 3 was considered a positive response.

The cytokine levels secreted by the T cells in the supernatants were quantified using OptEIA ELISA kits (BD, Pharmigen, San Diego, CA), according to manufacturer’s instructions. Briefly, each ELISA was performed using pairs of antibodies against IL-6, IL-1β, TNF-α, IL-10, IL-17A (IL-17), IL-21, GM-CSF and IFN-γ. The reaction was revealed with streptavidin–horseradish peroxidase, using 3,3’,5,5’-tetramethylbenzidine (TMB) as substrate. Recombinant human cytokines, at concentrations ranging from 3.5–500 pg/mL, were used to construct standard curves. The plates were read using Multiskan™ FC microplate photometer (Thermo Fischer Scientific).

### Statistical analyzes

The statistical analysis was performed using Prism 8.0 software (GraphPad Software). All immunological evaluations were performed in triplicate for each individual and the intra-assay variability ranged from 9.3% to 17.1% (median value of 10.1%) as calculated by the software above. Comparisons between immune assays in cell cultures from the three different groups were performed with ANOVA followed by Tukey test for data with Gaussian distribution and by Kruskal–Wallis followed by Dunn’s test for data without Gaussian distribution. The results were also corrected by Bonferroni. The nonparametric Mann–Whitney *U* test and the Student’s *t* test were applied to determine whether the two groups were statistically different for nonparametric and parametric variables, respectively. Significance in all experiments was *p* < 0.05.

## Results

### Activated effector CD4^+^ and CD8^+^ T cells expressing functional TLR2 and, mainly, TLR4 are expanded in symptomatic CCM patients

For this study peripheral blood was collected from 37 CCM patients, 14 asymptomatic (CCM_Asympt_, mean ± SD, 45 ± 13.1 years) and 23 symptomatic (CCM_Sympt_, mean ± SD, 40 ± 13.6 years) (Table 1). For control group (Ctrl), blood samples were obtained from 20 healthy subjects. As expected [3], the majority of CCM patients was female and the Sporadic form of disease was dominant. Out of the 23 symptomatic patients, 3 (13%) presented with epilepsy and the other 20 (87%) presented with at least one episode of bleeding. Among the 3 patients with epilepsy, 2 were under pharmacological treatment with oxcarbazepine at the time of blood sampling (Table 1). Despite the use of therapy to control epileptic seizures, these two patients were not excluded, because no difference was observed regarding immunological assays among them and the other symptomatic patients. Developmental venous anomaly (DVA) was identified in 03 asymptomatic and 09 symptomatic patients. The majority of patients and healthy subjects were Caucasians.

**Table 1:** Features and demographic of CCM patients and healthy subjects

Characteristics	CCM patients			Health non-CCM subjects
	Symptomatic		Asymptomatic	
	Bleeding	Epilepsy		
Sample size, *N* (%)	20 (55%)	3 (8%)^a^	14 (37%)	20
Age, years (mean)	44	38	48	40.1
Female, *N* (%)	12 (60%)	3 (100%)	10 (71%)	10 (50%)
Sporadic, *N* (%)	17 (85%)	3 (100%)	7 (50%)	NA^c^
Familiar, *N* (%)	3 (15%)	0	7 (50%)	NA
DVA, *N* (%)^b^	8 (40%)	1 (33%)	3 (21%)	NA
Ethnicity				
Caucasian. *N* (%)	18 (88%)	2 (66%)	9 (66%)	14 (70%)
African-Brazilian, *N* (%)	2 (12%)	1 (34%)	5 (34%)	6 (30%)

^a^Two from three patients with epilepsy were using Oxcarbazepine (Trileptal) to control seizure crises. The subjects, patients and healthy individuals recruited, were not taking any medication at the time of blood sampling. ^b^Developmental venous anomaly. ^c^Not applicable

**Fig. 1:**
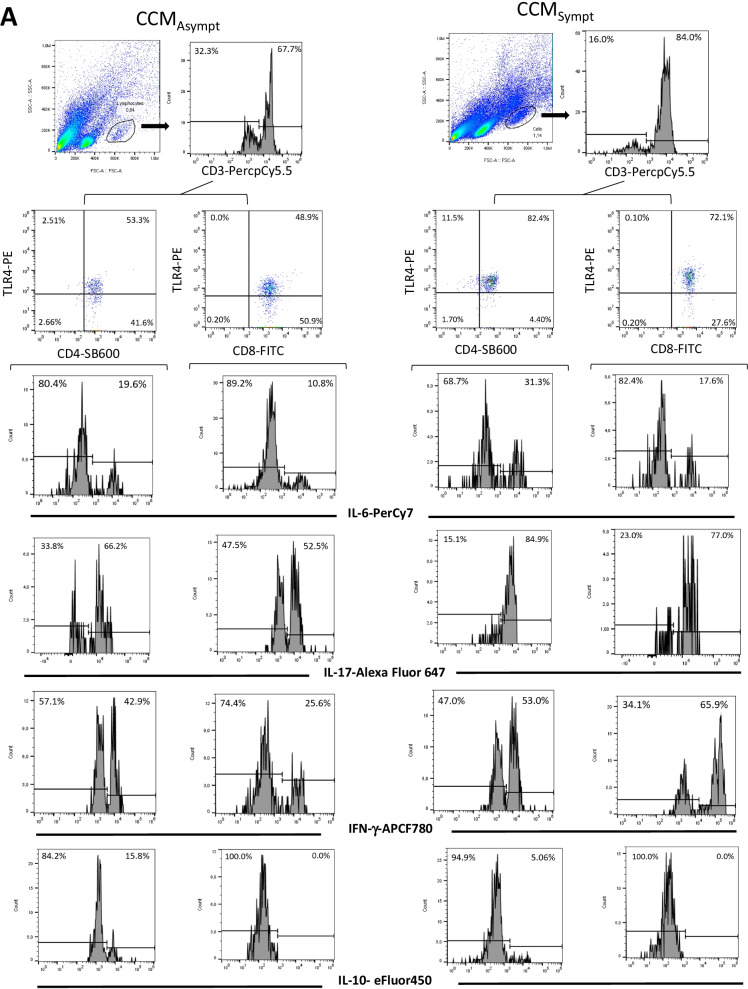

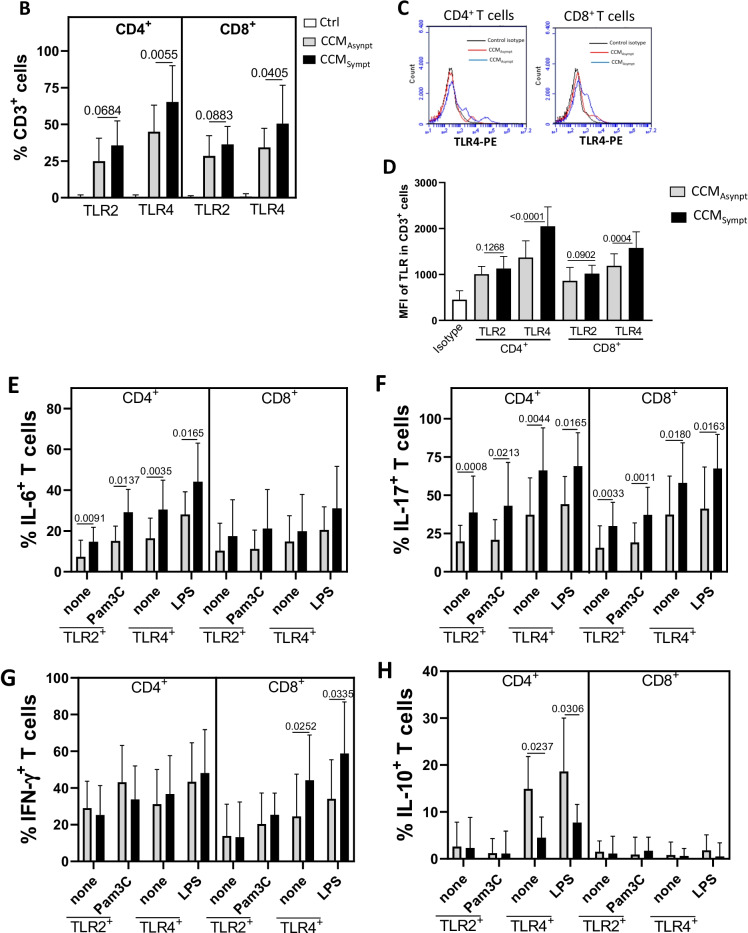
The frequency of cytokine-producing TLR2^+^ and TLR4^+^ T cells from CCM patients. In (**B**), the mean proportion of CD4^+^ and CD8^+^ T cells positive for TLR2 and TLR4, as well as (**D**) MFI of TLR2 and TLR4 for these cells, was determined by cytometry following representative dot-plots and histograms shown in figures **A** and **C** after acquisition of 200,000 to 300,000 events in samples obtained from asymptomatic (CCM_Asympt_, *n* = 14) and symptomatic (CCM_Sympt_, *n* = 23) CCM patients. The mean percentage of these cells positive for IL-6 (**E**), IL-17 (**F**), IFN-γ (**G**), and IL-10 (**H**) was determined by flow cytometry (figure **A**) in the absence of stimuli (none) or 24 h after activation with Pam3Csk4 (Pam3C, 1 μg/mL) or LPS (100 ηg/mL). Data are shown as mean ± SD of seven independent experiments with 5 to 6 samples per experiment. Significance was calculated by comparing different cell culture conditions from CCM_Asympt_ and CCM_Sympt_ groups, and the *p* values are shown in the figure (**B** and **D**, Ordinary ANOVA test and Turkey test; **E** to **H**, Kruskal-Wallis test and Dunn’s test).

The main objective of this study was to evaluate the expression of TLR2 and TLR4 on (CD4^+^ and CD8^+^) T lymphocytes from CCM_Asympt_ and CCM_Sympt_ patients, as well as the cytokine profile of these T cell subsets. Taking into account the FSC versus SSC parameters (Additional file 1: Fig. S1A), the frequency of (CD4^+^ and CD8^+^) T cells expressing TLR2 and TLR4 in the classical T cell gate was low and showed no difference between healthy subjects (control group) and CCM_Asympt_ or CCM_Sympt_ patients (Additional file 1: Fig. S1B). Furthermore, these T cells were negative for IL-6, IL-17, IFN-γ and IL-10 cytokines (data not shown). On the other hand, higher frequency of T cells expressing TLR2 and TLR4 was identified in the gate containing larger and more granular CD45^+^ cells in blood samples from CCM patients (Additional file 2: Fig. S2A). These T cells were negative for CD14 and CD16 markers, excluding the presence of monocytes and NK cells (Additional file 2: Fig. S2). Moreover, taking into account the expression of CD62L and CD45RO (Additional file 2: Fig. S2), the great majority of these larger CCM-derived T cells exhibited central memory (CD45R0^+^CD62L^+^) and effector memory (CD45R0^+^CD62L^−^) phenotypes (Additional file 2: Fig. S2B). Of note, these lager T cell subsets were almost absent in the control group, which made any additional immune analysis impossible (Additional file 1: Fig. S1 and Fig. 1B). Among CCM patients, using the gating strategy shown in Fig. 1A, the proportion of TLR4^+^ (CD4^+^ and CD8^+^) T cells was found to be significantly higher in CCM_Sympt_ patients (Fig. 1B). A similar tendency was observed for TLR2^+^ CD4^+^ T cells (*p* = 0.0684) and TLR2^+^ CD8^+^ T cells (*p* = 0.0883) (Fig. 1B). Taking into account the mean fluorescence intensity (MFI), the expression of TLR4 molecules per cell was significantly higher on (CD4^+^ and CD8^+^) T cells from CCM_Sympt_ patients (Fig. 1C, D).

**Fig. 2:**
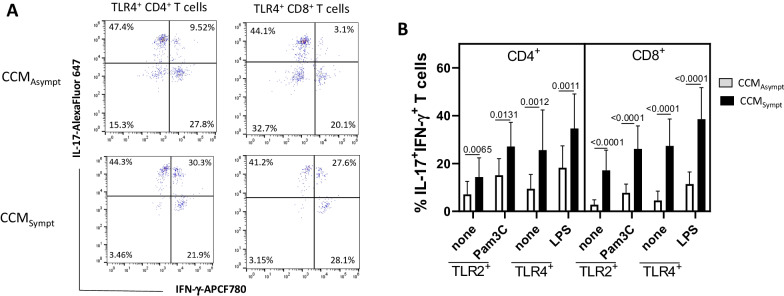
An expansion of IL-17^+^IFN-γ^+^ T cells positive for TLR2 and TLR4 was observed in symptomatic CCM patients. Taking into account the representative dot-plots shown in figure (**A**), the mean frequency of (**B**) dual IL-17 and IFN-γ-secreting CD4^+^ and CD8^+^ T cells positive for TLR2 and TLR4, before and after Pam3Csk4 (Pam3C, 1 μg/mL) or LPS (100 ηg/mL) addition, was determined by cytometry after acquisition of 200,000 to 300,000 events in samples obtained from asymptomatic (CCM_Asympt_, *n* = 14) and symptomatic (CCM_Sympt_, *n* = 23) CCM patients. Data are shown as mean ± SD of seven independent experiments with 5 to 6 samples per experiment. Significance was calculated by comparing different cell culture conditions from CCM_Asympt_ and CCM_Sympt_ groups, and the *p* values are shown in the figure (Kruskal-Wallis test and Dunn’s test)

Concerning the cell phenotypes, even without stimulation, a higher frequency of TLR2^+^ and TLR4^+^ CD4^+^ T cells able to produce IL-6 (Fig. 1E) and IL-17 (Fig. 1F) was detected in the CCM_Sympt_ group. Similarly, CCM_Sympt_ patients also showed a significantly higher percentage of both IL-17^+^CD8^+^ T cells, positive for TLR2 and TLR4 (Fig. 1F), and IFN-γ^+^ TLR4^+^ CD8^+^ T cells (Fig. 1G) than CCM_Asympt_ patients. On the other hand, CCM_Asympt_ group had a higher percentage of IL-10^+^TLR4^+^CD4^+^ T cells (Fig. 1H). From combinatorial analyses of different cytokines, we observed that the frequency of CD4^+^ and CD8^+^ T cells, expressing TLR2 or TLR4, able to simultaneously produce IL-17 and IFN-γ was significantly higher in CCM_Sympt_ than in CCM_Asympt_ patients (Fig. 2A, B), with no difference for dual positive cells for IL-17 and IL-6 (Additional file 3: Fig. S3A) or IFN-γ and IL-6 (Additional file 3: Fig. S3B). Unfortunately, as previously demonstrated, the extremely low frequency of these T cells in samples from control group (healthy subjects) made any further analysis of the cytokine profile impossible.

To determine if these TLRs are functional, the T cell subsets were reanalyzed after the addition of Pam3C and LPS. In general, CD4^+^ T cells were more responsive to TLR agonists than CD8^+^ T cells (Fig. 1E to H). Only in CD4^+^ T cell cultures Pam3C and LPS increased the frequency of IL-6^+^ cells, mainly among CCM_Sympt_ patients (Fig. 1E). Although neither Pam3C nor LPS changed the percentage of TLR^+^ (CD4^+^ and CD8^+^) T cells able to produce IL-17 (Fig. 1F) and IFN-γ (Fig. 1G), these TLR ligands significantly elevated the proportion of IL-17^+^IFNγ^+^ (CD4^+^ and CD8^+^) T cells in CCM_Asympt_ and, mainly, CCM_Sympt_ patients (Fig. 2). Finally, the proportion of IL-10^+^ TLR4^+^ CD4^+^ T cells was only up regulated by LPS in CCM_Asympt_ patients (Fig. 1H).

### Purified CD4^+^ and CD8^+^ T cells from symptomatic CCM patients were more responsive to stimulation via TCR/CD28 complex and ligands for TLR2 and TLR4

**Fig. 3:**
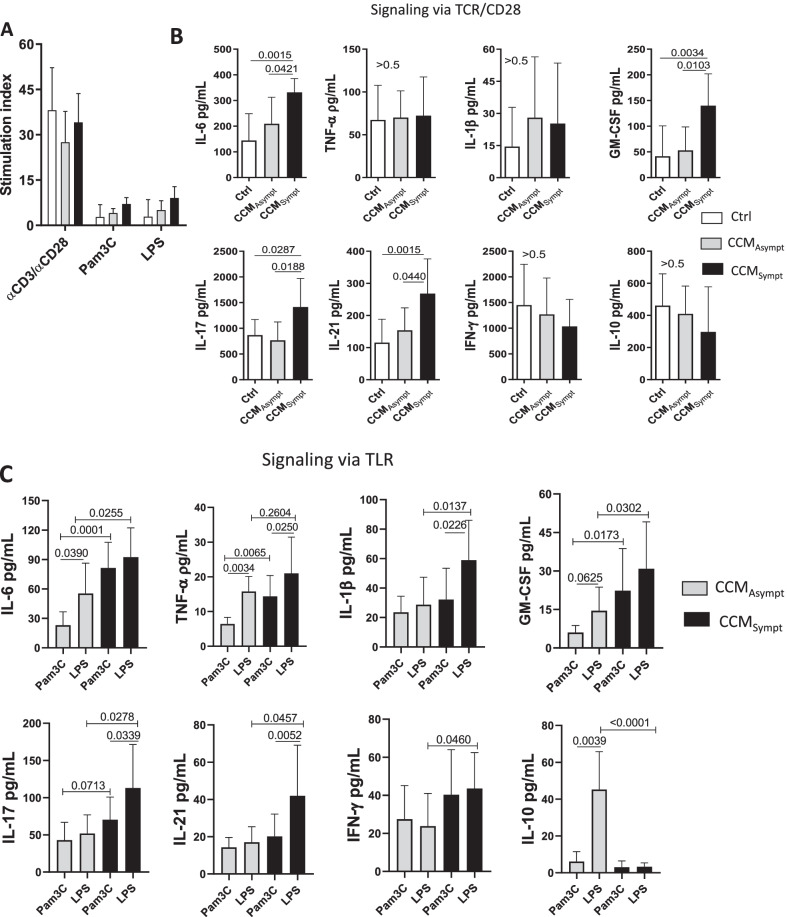
T cells from symptomatic CCM patients were more responsive to TLR ligands and TCR/CD28 activation. T cell cultures (1 x 10^6^/mL) from healthy subjects (Ctrl, *n* = 20) and CCM_Asympt_ (*n* = 10) and CCM_Sympt_ (*n* = 10) patients were maintained in the presence of anti-CD3/anti-CD28 beads (10 μL/mL) or with ligands for TLR2 (Pam3C, 1 μg/mL) and TLR4 (LPS, 100 ηg/mL). After 48 h, the (**A**) T cell proliferation was determined by [^3^H]TdR up take, and the cytokine release after activation via TCR/CD28 (**B**) or TLR ligands (**C**) was evaluated by ELISA. Data are shown as mean ± SD of ten independent experiments with 4 samples per experiment. Significance was calculated by comparing different cell culture conditions from Ctrl, CCM_Asympt_ and CCM_Sympt_ patients (IL-21 and GM-CSF, Kruskal-Wallis test and Dunn’s test; IL-17, IL-6, IFN-γ, IL-10, IL-1β and TNF-α, ordinary ANOVA test and Turkey test)

To verify whether T cells are directly responsive to TLR2 and TLR4 ligands, T cells from CCM patients were purified and cultured for 48 h with Pam3C and LPS. As a positive control, the cells were stimulated with anti-CD3/anti-CD28-coated beads. In addition, the same analysis was performed with T cell cultures from 20 healthy adult subjects. As demonstrated in Fig. 3A, no difference concerning T cell proliferation was observed between CCM patients (asymptomatic and symptomatic) and the control group in response to either CD3/CD28 mAbs or TLR ligands. With regard to cytokine release, T cell activation via TCR/CD28 complex induced higher production of IL-6, IL-17, GM-CSF and IL-21 in cell cultures from CCM_Sympt_ patients when compared with both CCM_Asympt_ patients and the control group (Fig. 3B). No difference was observed for TNF-α, IFN-γ and IL-10. Among TLRs, LPS was more potent than Pam3C in inducing the release of IL-6, TNF-α, IL-1β, GM-CSF, IL-17 and IL-21 by T cells in the CCM_Sympt_ group than in the CCM_Asympt_ group (Fig. 3C). IFN-γ levels were also higher in LPS-stimulated T cell cultures from CCM_Sympt_ patients (Fig. 3C). Similar to flow cytometry result, the release of IL-10 was significantly higher in LPS-activated T cells from CCM_Asympt_ patients (Fig. 3C). The levels of those cytokines in the supernatants from the control group were almost undetectable (data not shown).

### Elevated frequency of memory B cell subsets is observed in symptomatic CCM patients

**Fig. 4:**
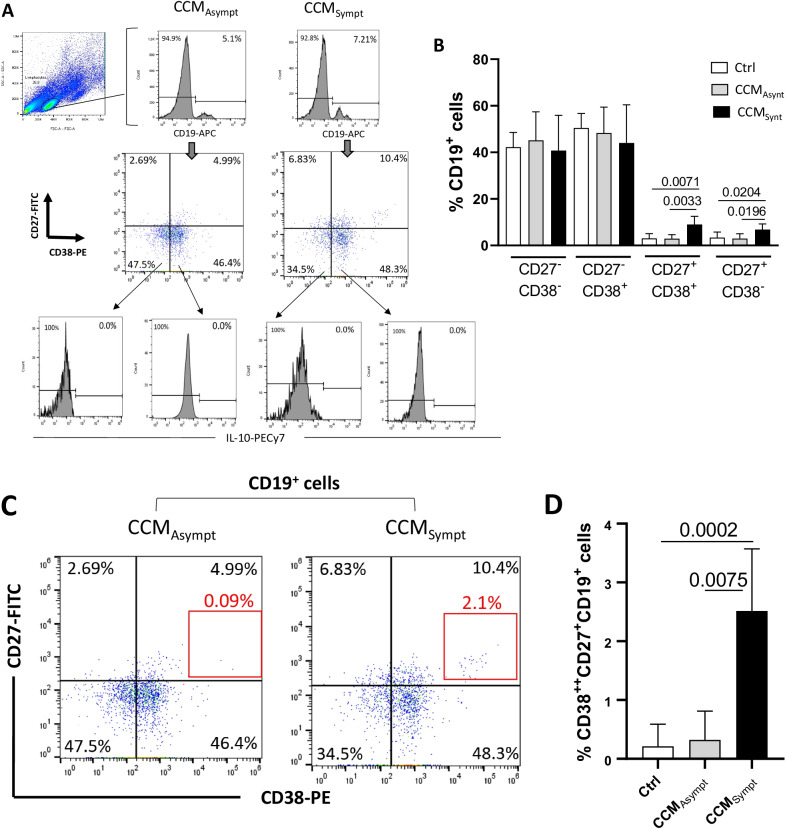

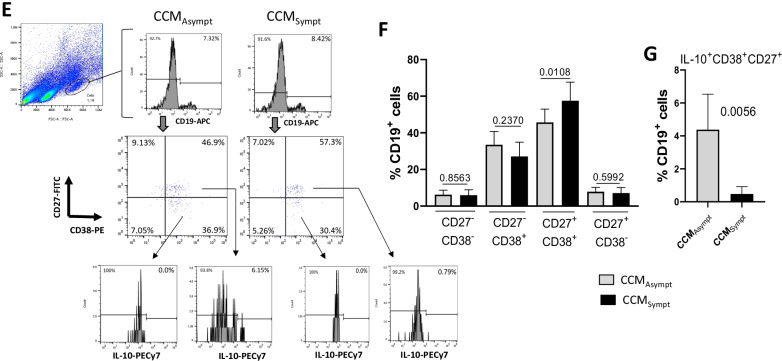
B cell subsets in CCM patients. Following the gate strategy and representative dot-plots and histograms shown in panels (**A**) and (**C**) we determined the mean frequency of different B cell subsets in healthy subjects (Control, Ctrl, *n* = 20) and patients [CCM_Asympt_ (*n* = 14) and CCM_Sympt_ (*n* = 23)] using a combination of monoclonal antibodies anti-CD19, anti-CD27, anti-CD38 and anti-IL-10. A similar analysis was performed among larger and more granular B cells in CCM patients (**E**–**G**). Data are shown as mean ± SD of 5 independent experiments with 4 to 7 samples per experiment. Significance was calculated by comparing different cell culture conditions from CCM_Asympt_ and CCM_Sympt_ groups (**B** and **D** Kruskal–Wallis and Dunn’s test; **D** to **F**, Student’s t test)

In addition to T cells, B cells and antibodies have been found in CCM lesions [6, 27, 28]. Using CD19, CD27 and CD38, we identified naïve (CD19^+^CD27^−^CD38^+^), memory (CD19^+^CD27^+^CD38^−^) and memory activated (CD19^+^CD27^+^CD38^+^) B cells [29–31]. Taking into account the gate strategy shown in Fig. 4A a higher frequency of CD19^+^CD27^+^CD38^−^, CD19^+^CD27^+^CD38^+^ cells (Fig. 4B), and CD19^+^CD27^+^CD38^+^ cells (Fig. 4C, D) was observed in CCM_Sympt_ patients as compared with control group and CCM_Asympt_ group. All these B cells were negative for IL-10 (Fig. 4A). No difference in the frequency of B cell subsets was observed between CCM_Asympt_ patients and control group (Fig. 4B, D).

Interestingly, even showing low frequency, the percentage of CD19^+^CD27^+^CD38^+^ in larger and more granular cell gate was significantly higher in samples from CCM_Sympt_ than CCM_Asympt_ patients (Fig. 4E, F). In contrast, the proportion of these cells able to produce IL-10 was significantly higher in CCM_Asympt_ patients (Fig. 4G). Of note, these cells were negative for CD14, and very low frequency were positive for CD16 (2.78% ± 1.96%) (Additional file 1: Fig. S2A).

## Discussion

CCMs are vascular lesions consisting mainly of clusters of brain capillaries that are grossly dilated and present altered permeability. This predisposes patients to greater risk of hemorrhagic stroke, epilepsy and various neurological deficiencies [1]. Despite the knowledge about the genes involved in the disease, severity and outcomes of CCM depends on the activation of MEKK3-KLF2/4 pathway by external triggers [32], such as inflammatory mediators. In CCM, elevated plasma levels of IL-1β, IL-2, IFN-γ and TNF-α have been associated with disease activity [11, 33], probably by activating the MEKK3-KLF2/4 pathway [8, 9]. Interestingly, in murine model of CCM, the severity of the disease was associated with production of pro-inflammatory cytokines by endothelial cells simulated via CD14/TLR4 pathway by LPS [14]. Although the role of this PAMP in the experimental model of CCM did not depend on local immune cells, abundant innate and specific immune cells have been detected in the brain lesions in patients. Although resting T cells express very low TLR levels, these pattern receptors are strongly expressed in chronically-activated T cells associated with severity of multiple sclerosis (MS) and neuromyelitis optica spectrum disorder (NMOSD), both autoimmune diseases of the CNS [22, 24, 34, 35].

In the present study, CCM_Sympt_ patients (87% with lesional hemorrhage and 13% with seizure crisis) had a higher frequency of (CD4^+^ and CD8^+^) T lymphocytes expressing TLR4 gated in a region containing larger and more granular cells than CCM_Asympt_ patients. This region was absent in the samples of the control group. Among the patients, the intensity of TLR4 molecule expression per (CD4 + and CD8 +) T cell was also higher in symptomatic individuals. Corresponding with their activated state, these cells were positive for some pro-inflammatory cytokines, and CCM_Sympt_ patients presented a higher proportion of IL-6^+^CD4^+^ T cells, Th17-like cells and Tc-17-like cells IL-17 cells expressing TLR2 and TLR4 than CCM_Asympt_ patients. In addition, the symptomatic group had a higher proportion of TLR4^+^ Tc-1-like cells. Interestingly, clinical activity of CCM was associated with an elevated percentage of dual IL-17^+^ and IFN-γ^+^ (CD4^+^ and CD8^+^) T cells expressing TLR2 and TLR4. Furthermore, the addition of Pam3C and LPS to the cell cultures increased, mainly in CCM_Sympt_ patients, the percentage of TLR2^+^ and TLR4^+^ CD4^+^ T cells capable of producing IL-6 and Th17.1 cells. Preliminary analysis did not show any significant correlation between the frequency of cytokine-producing TLR^+^ T cell subsets and brain lesion location (data not shown). Due to financial limitations, it was not possible to evaluate the expression of other TLRs in combination with different cytokines. In line with our findings, elevated frequency of TLR^+^ Th17 cell subsets has been associated with MS and NMOSD [24, 26, 36–38].

The relationship between increased frequency of Th17 cell subsets, which classically express CCR6, and CCM clinical activity is notable, since the entry of T cells in the CNS is facilitated by the choroid plexus, a region with weak blood–brain barrier and high expression of CCL20, natural ligand of CCR6 [24, 39, 40]. In CCM areas, the local production of IL-17 and IFN-γ by Th17.1 cells would activate other local (microglia) and migrant (monocytes and B cells) immune cells, as well as endothelial cells, which increase the tissue damage. If reproduced in a larger sample size, these findings would indicate the possible involvement of autoimmune components in CCM prognosis.

Besides the expansion of pathogenic Th17 cells, autoimmune diseases are classically associated with impaired production of the anti-inflammatory cytokine IL-10 by regulatory CD4^+^ T cells [41–45]. In fact, an inverse relationship between the quantity of iron accumulated in the CCM and IL-10 was reported after a symptomatic hemorrhage [10]. Similarly, in our study, lower percentages of IL-10^+^TLR4^+^CD4^+^ T cells were observed in CCM_Sympt_ patients. Among the TLR ligands, LPS, but not Pam3C, enhanced both the proportion of IL-10^+^TLR4^+^CD4^+^ T cells and IL-10 released by purified T cells only in asymptomatic patients.

It is known that LPS responsiveness is amplified by CD14, a molecule expressed on monocyte surfaces [12, 13]. Gene polymorphisms expected to increase the expression of both membrane CD14 (mCD14) and TLR-4 have been correlated with more severe CCM disease [10, 11]. The mCD14/TLR4, when complexed to LPS, triggers intracellular signaling cascades that upregulate the production of pro-inflammatory cytokines [12, 13]. In the present study, although TLR2^+^ and TLR4^+^ T cells from CCM patients were negative for CD14, these cells responded directly to Pam3C and LPS. Here, the release of IL-6, TNF-α, IL-1β, IL-17, GM-CSF and IL-21 by purified T cells was significantly higher in CCM_Sympt_ than in CCM_Asympt_ patients. In MS patients, the release of IL-6, IFN-γ, IL-17 and GM-CSF by Pam3C-activated CD4^+^ T cells was directly associated with disease activity [24]. A similar clinical correlation was observed for IL-17 levels released by Pam3C-stimulated CD8^+^ T cell cultures from MS patients [40]. Rajaiah et al. [46] demonstrated that while CD14 is essential for LPS-induced TLR4 activation of TRIF-mediated signaling in macrophages, this accessory molecule is not required for MyD88-mediated signaling via LPS/TLR4 complex, allowing MAPK and NF-κB activation, and TNF-α and IL-6 production. In addition, soluble CD14 (sCD14) can be involved in CCM-derived T cells responsiveness to LPS signaling in cells lacking membrane-bound CD14. Thus, the lower sCD14 levels in CCMSympt patients observed by some authors [33] may be explained by an uptake of LPS/sCD14 complex by TLR4+ T cells.

In light of the discussed above, clinical conditions associated with high circulating concentrations of LPS, as well as other PAMPs, can be triggering factors for disease activity. Interestingly, gut dysbiosis with overgrowth of GNB species has been associated with clinical activity of CCM [47–49]. Furthermore, endogenous TLR ligands, named danger-associated molecular patterns (DAMPs), could also contribute to the disease. Some of these DAMPs are metabolic products from the destruction of extracellular matrix by matrix metalloproteinase-2 (MMP2) produced by activated immunity cells in CCM lesions [50, 51]. Other DAMPs, such as the intracellular protein HMGB, by activating immune cells through TLR2 and TLR4 [52, 53], could also contribute to CCM pathogenesis.

Besides TLR signaling, the cytokine profile of TCR/CD28-activated T cells from CCM patients in our study was also different as an expression of clinical activity. In this context, elevated production of IL-6, IL-17 and IL-21 by purified T cells from CCM_Sympt_ individuals was observed as compared with healthy subjects and asymptomatic patients. Concerning the cytokine profile by mitogen-activated T cells, no difference between CCM_asympt_ and control group. These results are interesting, because pathogenic GM-CSF^+^Th17 cell subsets, by promoting recruitment and activation of peripheral dendritic cells and monocytes, are implicated in MS pathogenesis [54–56], and this phenotype can also contribute to the infiltration of these phagocytes into CCM areas. Furthermore, elevated release of IL-21 and IL-6 by CCM_Sympt_-derived T cells in response to both TLR4 ligand and TCR/CD28 signaling should contribute to local IgG production.

In addition to IgG deposits, studies by Shi et al. demonstrated the presence of well-organized clusters of CD4^+^ T cells, B cells, and plasma cells, which are found in CCM lesions [6, 28]. To date, it is known that somatically hypermutated IgG strictly depends on T_FH_ cells, a newly identified CD4^+^ T cell subset capable of producing IL-21 and IL-6 [39], both cytokines implicated in B cell activation, generation of memory B cells and long-lived plasma cells in lymph nodes [15, 57]. The involvement of T_FH_ cells needs to be investigated, since the activation of T_FH_/B cell axis could induce CCM lesions mediated by local complement activation and in situ IgG production, as described by Shi et al. [6].

With regard to B cell subsets, a higher proportion of memory (CD27^+^CD38^−^) and memory activated (CD27^+^CD38^+^ and CD27^+^CD38^++^) B cells was detected in CCM_Sympt_ than in CCM_Asympt_ patients and healthy subjects. Notably, an expansion of the CD27^+^CD38^++^ B cell subset was identified as a biomarker for relapse prediction in anti-neutrophil cytoplasmic autoantibody (ANCA)-associated vasculitis (AAV) [29], humoral autoimmune diseases involving small to medium-sized blood vessels [1]. Therefore, there is a possibility of involvement of the CD38^++^ B cell subset in CCM pathogenesis. Indeed, CCM_Sympt_ patients presented a higher proportion of memory B lymphocytes, among larger and granular cells, than CCM_Asympt_ patients. In contrast, the proportion of these cells able to produce IL-10 was significantly higher in CCM_Asympt_ patients.

Although our findings are interesting, this study has some limitations. First, this is a cross-sectional study with results based on cell cultures. A prospective study design could allow to draw conclusions about the causality between the frequency of different T and B cell subsets and the risk of new clinical events among CCM patients. Moreover, the involvement of other TLRs and their ligands needs to be investigated.

## Conclusions

In summary, our results reveal an expansion of effector TLR2^+^ and, mainly, TLR4^+^ T cells able to produce pro-inflammatory cytokines related to Th17 and Tc-17 phenotypes in symptomatic CCM patients, in association with a decrease in the frequency of IL-10-secreting TLR4^+^ T cells. A similar cytokine profile was observed in TCR/CD28-activated T cells from those patients. Furthermore, expansion of memory B cell subsets was also observed in patients with clinically active CCM.

## Supplementary Information

**Additional file 1: Fig. S1.** Percentage of TLR4^+^ T cells in healthy subjects (n = 20) and asymptomatic (n = 14) and symptomatic (n = 23) CCM patients. In **B**, the mean percentage of TLR-4^+^ (CD4^+^ an CD8^+^) T cells from each experimental group was determined and significance calculated by comparing different experimental groups (Kruskal–Wallis test and Dunn’s test).

**Additional file 2: Fig. S2.** Phenotypic characterization of T cells, expressing TLR, and B cells gated on larger and more granular cells from CCM patients. In (**A**), the gating strategy and identification of different larger and more granular lymphocytes according to the expression of CD3, CD19, CD14, CD16 and CD45RO markers. In (**B**), the mean percentage of naïve (CDR5RO^−^CD62L^+^), central memory (CM, CDR5RO^+^CD62L^+^), effector memory (EM, CDR5RO^+^CD62L^−^) and terminally differentiated effector memory (TEMRA, CD45RO^−^CD62L^−^) T cells from CCM patients was determined and significance calculated by comparing the proportion of different T cell phenotypes according CD45RO and CD62L expression (Kruskal–Wallis test and Dunn’s test).

**Additional file 3: Fig. S3.** Frequency of IL-17^+^IL-6^+^ and IL-6^+^IFN-γ^+^ T cells expressing TLR2 and TLR4 in CCM patients. The mean frequency of dual (**A**) IL-17 and IL-6- or (**B**) IL-17 and IFN-γ-secreting (CD4^+^ and CD8^+^) T cells positive for TLR2 and TLR4 from asymptomatic (CCM_Asympt_, n = 14) and symptomatic (CCM_Sympt_, n = 23) patients was determined before and after Pam3Csk4 (Pam3C, 1 μg/mL) or LPS (100 ηg/mL) addition. Data are shown as mean ± SD of seven independent experiments with 5 to 6 samples per experiment. Significance was calculated by comparing different cell culture conditions from CCM_Asympt_ and CCM_Sympt_ groups, and the *p* values are > 0.05 (Kruskal–Wallis test and Dunn’s test).

## Data Availability

All data generated or analyzed during this study are included in this article and its supplementary material. Further enquiries can be directed to the corresponding author.

## Abbreviations

CASH: Cerebral cavernous angioma with symptomatic hemorrhage
CCM: Cerebral cavernous malformations
CCM_Asympt_: Asymptomatic cerebral cavernous malformations
CCM_Sympt_: Symptomatic cerebral cavernous malformations
CNS: Central Nervous System
DAMPs: Danger-associated molecular patterns
EDSS: Expanded disability status scale
GD: Gut dysbiosis
GM-CSF: Granulocyte Macrophage Colony-Stimulating Factor
GNB: Gram-negative bacteria
GPB: Gram-positive bacteria
IFN-γ: Interferon gamma
IL: Interleukin
IL-1R: IL-1 receptor
LPS: Lipopolysaccharides
MAPK: Mitogen-activated protein kinase
mCD14: Membrane CD14
MMP-2: Matrix by matrix metalloproteinase-2
MS: Multiple sclerosis
MyD88: Myeloid differentiation primary response protein 88
NF-κB,: Nuclear factor-κB
NMOSD: Neuromyielitis optic spectrum disorders
PAMP: Pathogen-associated molecular pattern
PBMC: Peripheral blood mononuclear cells
sCD14: Soluble CD14
SI: Stimulation indexes
T_FH_: Follicular helper T
TLR: Toll-like receptor
TNF: Tumor necrosis factor
