# Supplementary figures and images for: Elevated proportion of TLR2- and TLR4-expressing Th17-like cells and activated memory B cells was associated with clinical activity of cerebral cavernous malformations
Source: J Neuroinflammation. 2022 Feb 2;19:28. doi: 10.1186/s12974-022-02385-2 (PMC8808981; doi:10.1186/s12974-022-02385-2)

## Slide 1
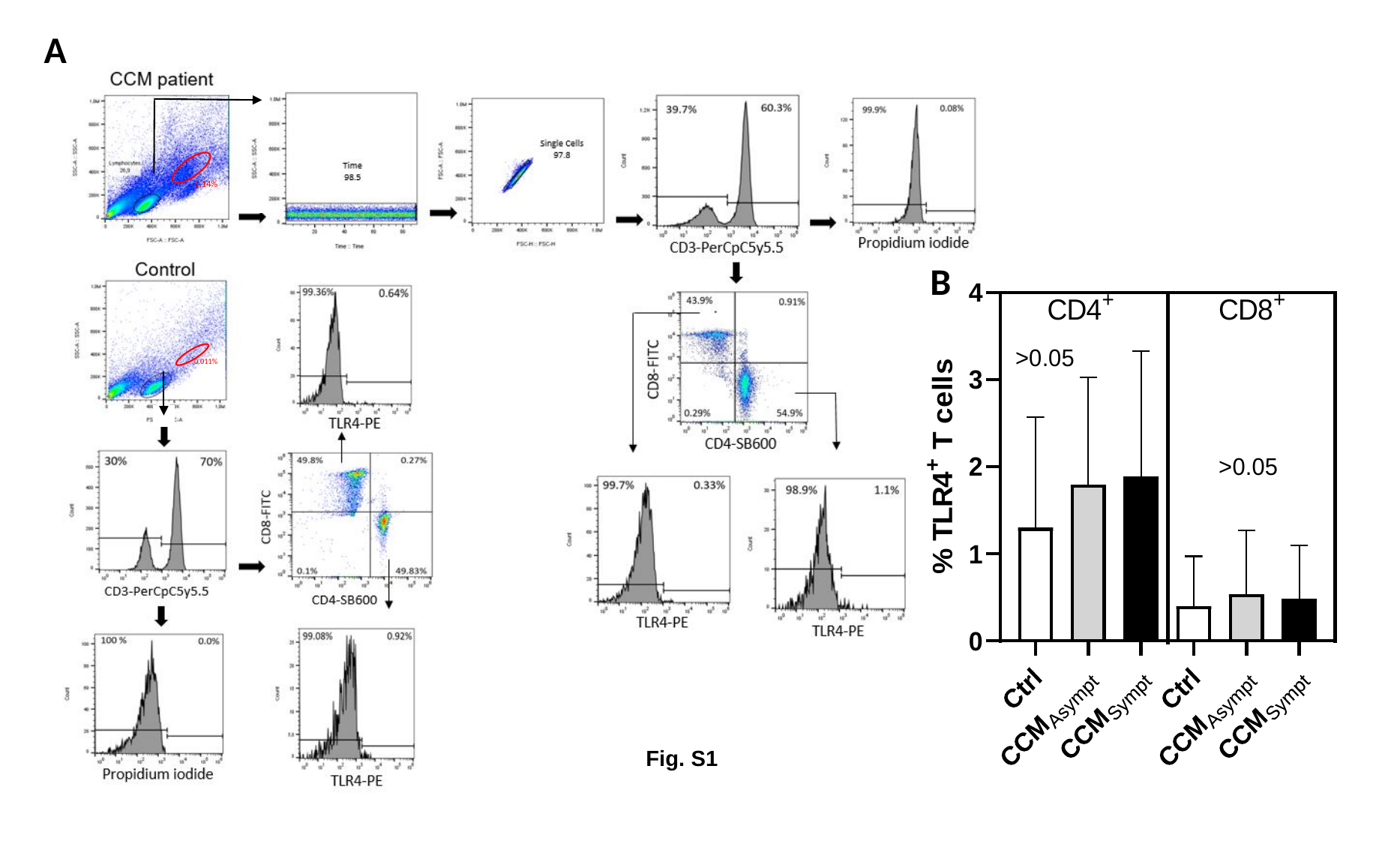

A
1.14%
B
0.011%
Fig. S1

Supplement: Supplementary file 1 — Additional file 1: Fig. S1. Percentage of TLR4+ T cells in healthy subjects (n = 20) and asymptomatic (n = 14) and symptomatic (n = 23) CCM patients. In B, the mean percentage of TLR-4+ (CD4+ an CD8+) T cells from each experimental group was determined and significance calculated by comparing different experimental groups (Kruskal–Wallis test and Dunn’s test). [file 12974_2022_2385_MOESM1_ESM.pptx]

## Slide 1
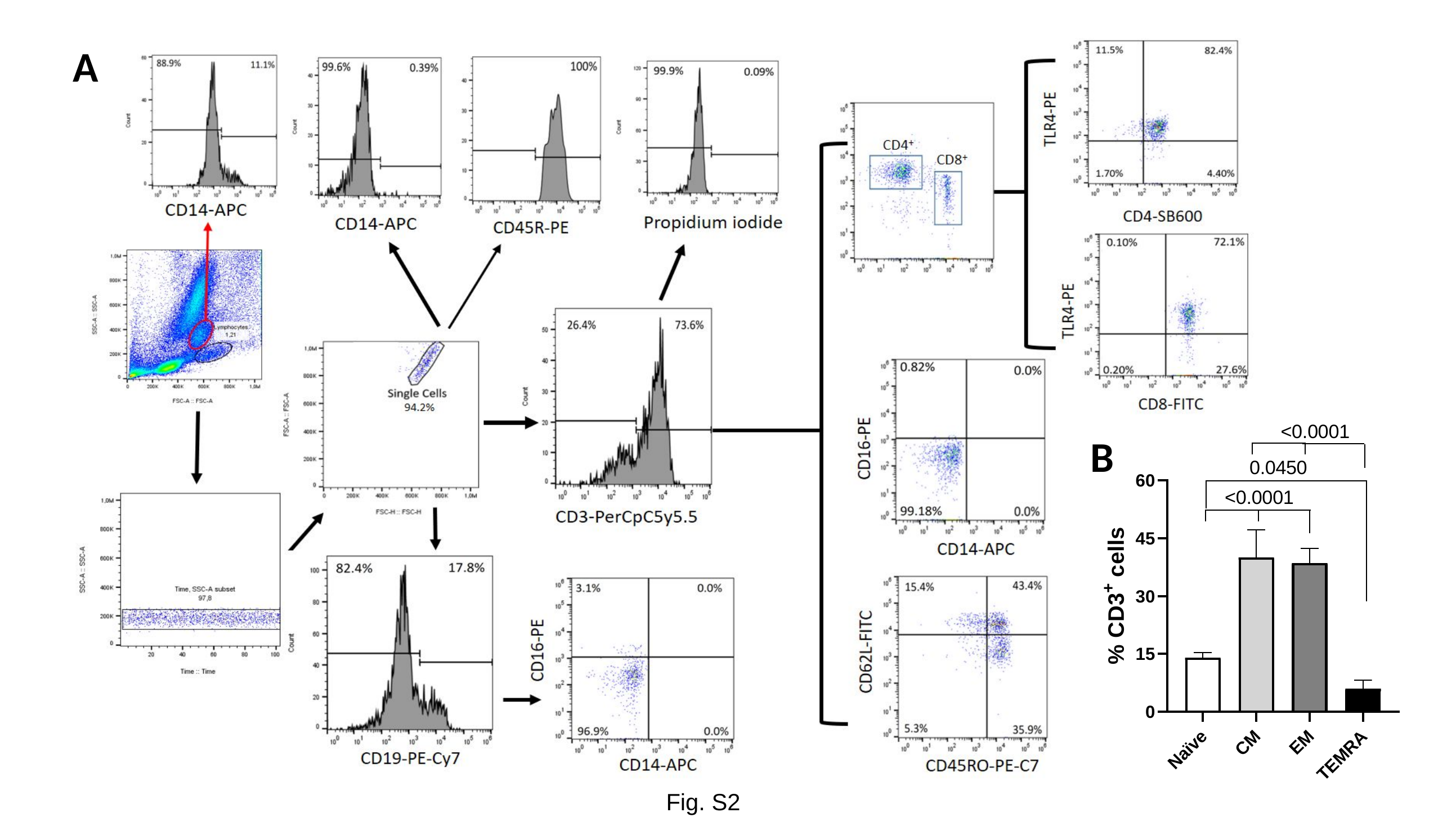

A
B
Fig. S2

Supplement: Supplementary file 2 — Additional file 2: Fig. S2. Phenotypic characterization of T cells, expressing TLR, and B cells gated on larger and more granular cells from CCM patients. In (A), the gating strategy and identification of different larger and more granular lymphocytes according to the expression of CD3, CD19, CD14, CD16 and CD45RO markers. In (B), the mean percentage of naïve (CDR5RO−CD62L+), central memory (CM, CDR5RO+CD62L+), effector memory (EM, CDR5RO+CD62L−) and terminally differentiated effector memory (TEMRA, CD45RO−CD62L−) T cells from CCM patients was determined and significance calculated by comparing the proportion of different T cell phenotypes according CD45RO and CD62L expression (Kruskal–Wallis test and Dunn’s test). [file 12974_2022_2385_MOESM2_ESM.pptx]

## Slide 1
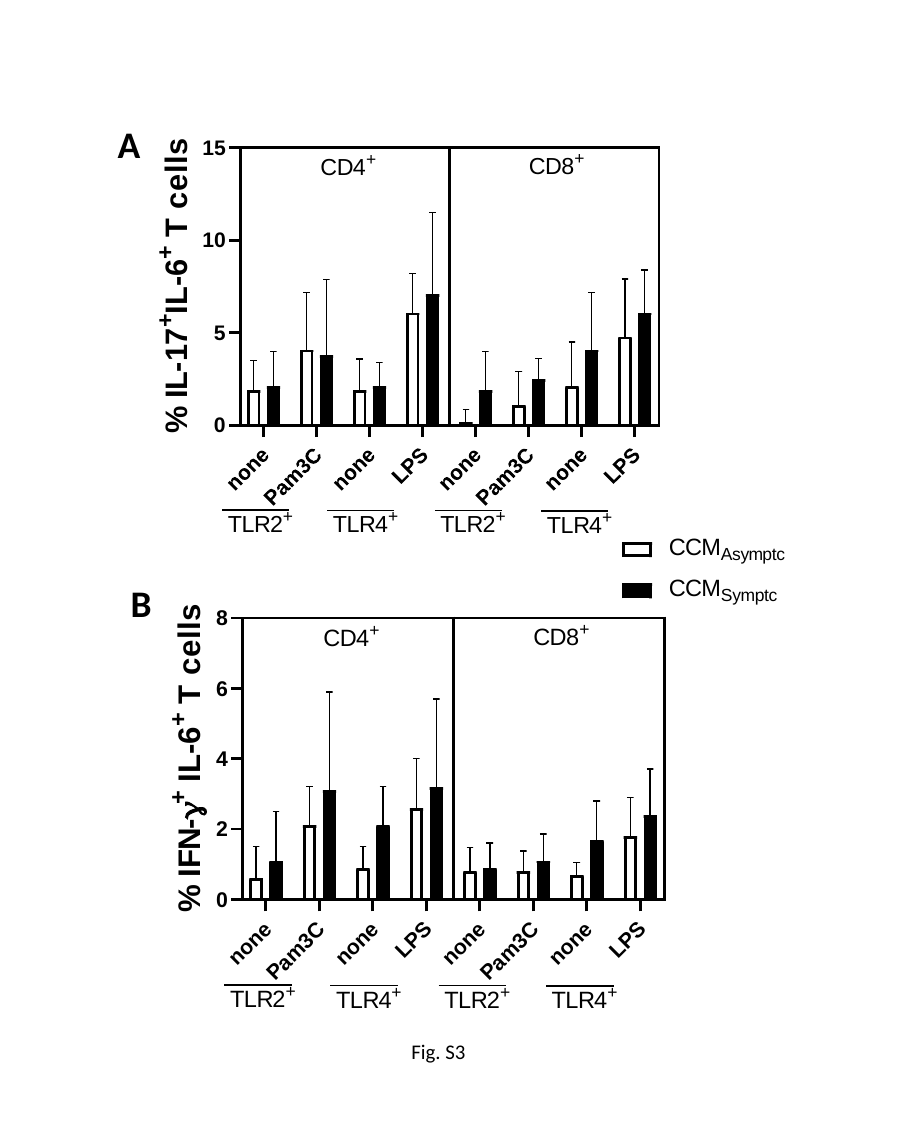

A
B
Fig. S3

Supplement: Supplementary file 3 — Additional file 3: Fig. S3. Frequency of IL-17+IL-6+ and IL-6+IFN-γ+ T cells expressing TLR2 and TLR4 in CCM patients. The mean frequency of dual (A) IL-17 and IL-6- or (B) IL-17 and IFN-γ-secreting (CD4+ and CD8+) T cells positive for TLR2 and TLR4 from asymptomatic (CCMAsympt, n = 14) and symptomatic (CCMSympt, n = 23) patients was determined before and after Pam3Csk4 (Pam3C, 1 μg/mL) or LPS (100 ηg/mL) addition. Data are shown as mean ± SD of seven independent experiments with 5 to 6 samples per experiment. Significance was calculated by comparing different cell culture conditions from CCMAsympt and CCMSympt groups, and the p values are > 0.05 (Kruskal–Wallis test and Dunn’s test). [file 12974_2022_2385_MOESM3_ESM.pptx]
